# Function of Torsin AAA+ ATPases in Pseudorabies Virus Nuclear Egress

**DOI:** 10.3390/cells9030738

**Published:** 2020-03-17

**Authors:** Julia E. Hölper, Barbara G. Klupp, G. W. Gant Luxton, Kati Franzke, Thomas C. Mettenleiter

**Affiliations:** 1 Institute of Molecular Virology and Cell Biology, Friedrich-Loeffler-Institut, 17493 Greifswald-Insel Riems, Germany; julia.hoelper@fli.de (J.E.H.); barbara.klupp@fli.de (B.G.K.); 2 Department of Genetics, Cell Biology and Development, University of Minnesota, Minneapolis, MN 55455, USA; gwgl@umn.edu; 3 Institute of Infectology, Friedrich-Loeffler-Institut, 17493 Greifswald-Insel Riems, Germany; kati.franzke@fli.de

**Keywords:** herpesvirus, pseudorabies virus, nuclear egress, AAA+ ATPase, Torsin, CRISPR/Cas9

## Abstract

Newly assembled herpesvirus nucleocapsids traverse the intact nuclear envelope by a vesicle-mediated nucleo-cytoplasmic transport for final virion maturation in the cytoplasm. For this, they bud at the inner nuclear membrane resulting in primary enveloped particles in the perinuclear space (PNS) followed by fusion of the primary envelope with the outer nuclear membrane (ONM). While the conserved viral nuclear egress complex orchestrates the first steps, effectors of fusion of the primary virion envelope with the ONM are still mostly enigmatic but might include cellular proteins like SUN2 or ESCRT-III components. Here, we analyzed the influence of the only known AAA+ ATPases located in the endoplasmic reticulum and the PNS, the Torsins (Tor), on nuclear egress of the alphaherpesvirus pseudorabies virus. For this overexpression of wild type and mutant proteins as well as CRISPR/Cas9 genome editing was applied. Neither single overexpression nor gene knockout (KO) of TorA or TorB had a significant impact. However, TorA/B double KO cells showed decreased viral titers at early time points of infection and an accumulation of primary virions in the PNS pointing to a delay in capsid release during nuclear egress.

## 1. Introduction

Herpesviruses are double-stranded DNA viruses, which use the host cell nucleus and the cytoplasm for replication and morphogenesis. While transcription, DNA replication, assembly of viral capsids as well as viral genome encapsidation take place in the nucleus, the nucleocapsid has to be transferred to the cytoplasm for final virion maturation. With a diameter of approximately 125 nm, its size far exceeds the 40 nm threshold for passage through intact nuclear pores [1]. However, no evidence for a significant impairment or alteration of barrier and gating functions of nuclear pores was found even at late time points after infection [2] demonstrating continued integrity of the nuclear envelope.

In eukaryotic cells, the nuclear envelope (NE) separates the nuclear contents from the cytoplasm. It consists of two concentric lipid bilayers designated as the inner (INM) and outer nuclear membrane (ONM) which are separated by the perinuclear space (PNS). The PNS is contiguous with the lumen of the endoplasmic reticulum (ER) as is the ONM with the ER membrane. In contrast, the INM harbors a unique set of membrane proteins distinct from that of the ONM and ER. INM and ONM are fused at sites where nuclear pore complexes (NPCs) are inserted, which also allow the import of the herpesviral genome at early stages of infection. Traffic into and out of the nucleus is thought to occur exclusively through NPCs (reviewed in Adam [3], Knockenhauer and Schwartz [4]). Interestingly, herpesvirus nucleocapsids are translocated through the nuclear envelope (NE) by a vesicle-mediated process designated as nuclear egress (reviewed in [5,6,7]).

Budding of herpesvirus nucleocapsids at the INM is driven by the nuclear egress complex (NEC) composed of two conserved herpesviral proteins designated as pUL31 and pUL34 in the alphaherpesviruses pseudorabies virus (PrV) and herpes simplex viruses (HSV-1, -2) [5,6,7]. The NEC is not only required for efficient nuclear egress, thereby generating primary enveloped virions in the PNS, but also sufficient for vesicle formation and scission from artificial lipid membranes and the INM, [8,9,10,11]. In a subsequent step, this primary envelope fuses with the ONM to release the nucleocapsids into the cytoplasm (reviewed in [12]).

Budding of nucleocapsids at the INM is quite well understood at the molecular level, while the fusion process of the primary envelope with the ONM remains mostly enigmatic. In contrast to reports for HSV-1 [13], the viral fusion machinery which is active during entry of herpesviruses is not involved in nuclear egress of PrV [14]. In addition, a variety of different PrV gene deletion mutants studied so far showed no detectable effect on nuclear egress arguing against a virus-encoded fusion machinery active at the NE. Only mutants lacking the alphaherpesvirus specific protein kinase pUS3 showed an impairment of nuclear translocation. In the absence of pUS3 [15,16,17] or by impairment of its kinase function [18,19,20] primary enveloped virions accumulate in herniations of the INM. However, pUS3 is not essential for viral replication and viral titers are only approx. 10-fold reduced. Based on these data, we speculated that herpesviruses might use a cellular machinery either already present in or recruited to the NE.

Although vesicle-mediated transport processes between cytoplasmic organelles and the plasma membrane are well studied, knowledge on vesicular transport and fusion events at the NE is poor. An example of such a cellular mechanism would be the fusion of the INM with the ONM that occurs during NPC insertion in a growing interphase nucleus (reviewed in Otsuka and Ellenberg [21]). This process is thought to involve an inside-out extrusion of the INM into and across the PNS followed by its subsequent fusion with the ONM [22,23]. Although the molecular mechanism underlying INM-ONM fusion remains incompletely understood, several cellular proteins have been implicated in this process including the multi-subunit endosomal sorting complex required for transport III (ESCRT-III) and the ATPase-associated with various cellular activities (AAA+) protein Vps4 (reviewed in Otsuka and Ellenberg [21]). Recent work suggests that ESCRT-III and Vps4 are also important for herpesvirus nuclear egress [24,25,26], but conflicting findings have been reported [27,28].

Two other potential cellular mediators of INM-ONM fusion during interphase NPC insertion are Torsin A (TorA) and TorB. As similar to Vps4, Torsins belong to the AAA+ ATPase superfamily. They function as molecular chaperones, which use energy derived from ATP-hydrolysis to remodel their target molecules and are involved in numerous processes including budding and fission of vesicles, and assembly as well as disassembly of protein complexes [29,30,31,32,33,34,35]. Torsins are composed of a N-terminal signal peptide, followed by a hydrophobic stretch (TorA and TorB) upstream of the Walker A and B motifs, which mediate ATP binding and hydrolysis [32]. TorA (*TOR1A*) and TorB (*TOR1B*) are atypical AAA+ proteins for the following three reasons. First, they contain a non-canonical Walker A motif [36]. Second, they and the related Tor2 (*TOR2A*), Tor3 (*TOR3A*), and Tor4 (*TOR4A*) proteins are the only AAA+ proteins known to reside within the contiguous ER lumen and PNS of the nuclear envelope [32,37,38,39]. Third, they lack the conserved ATP-hydrolysis-promoting arginine finger [35,40]. Consistent with the lack of an arginine finger, purified Tor proteins are unable to hydrolyze ATP in vitro [41]. Instead, they need to be activated by the direct interaction with the luminal domain of one of two known regulatory protein cofactors: the INM lamina-associated polypeptide 1 (LAP1) or the ER/ONM protein luminal domain-like LAP1 (LULL1). Mutations in TorA lead to an autosomal dominant disease in humans, called early-onset torsion dystonia 1 (DYT1/TOR1A dystonia) [42]. The most frequent disease linked form TorA_∆E302/303_ lacks a single glutamic acid residue at position Glu_302_ or Glu_303_ in the C-terminus of the protein [43,44,45]. None of the other Torsins are implicated in human disease. Nonetheless, EQ mutations in the Walker B domain of TorA or TorB lead to expression of ATP hydrolysis-deficient Torsin molecules [33,45] which exert dominant-negative effects [38,46,47]. While the substrates of TorA and TorB remain unknown, they and the related proteins Tor2 and Tor3 appear to function in a partially redundant manner [48,49,50]. Furthermore, in previous studies, TorA has been shown to play a role in NE maintenance. Specifically, severe defects in NE architecture with “blebbing” of the INM in neuronal tissue are observed in knockout (KO) mice or mice lacking proper Torsin function, by expression of the dystonia-related allele TorA_ΔE302/303_ [37,45,50,51,52,53]. This phenotype is reminiscent of the INM herniations, which were observed in cells overexpressing the NEC components [8] or in cells infected with US3-deletion mutants [15,16,17].

Consistent with a potential role during herpesvirus nuclear egress, overexpression of TorA or TorB resulted in slightly reduced HSV-1 titers in neuron-like and epithelial cells, as well as in the appearance of primary enveloped virions in cytoplasmic vesicles [54]. Moreover, HSV-1 replication was reduced in HeLa cells lacking both TorA and TorB [55]. To date, the molecular mechanism underlying the contribution of TorA and TorB to herpesvirus nuclear egress as well as interphase NPC biogenesis remains poorly defined. Nevertheless, a growing body of evidence supports the hypothesis that TorA is required for the assembly of functional linker of nucleoskeleton and cytoskeleton (LINC) complexes [39,48,56,57,58,59]. This conserved NE-spanning molecular bridge is present in all nucleated cells [60,61] and mechanically integrates the nucleus with the cytoskeleton mediating several fundamental cellular processes including cell division, DNA damage repair, meiotic chromosome pairing, mechano-regulation of gene expression, and nuclear positioning (reviewed in Meinke and Schirmer [62]).

LINC complexes are composed of ONM Klarsicht/ANC-1/SYNE homology (KASH)-domain and INM Sad1/UNC-84 homology (SUN)-domain containing proteins [63,64]. Although the LINC complex is involved in many essential cellular processes, it is still unknown how assembly and disassembly is achieved. TorA is reported to have affinity for the KASH domains of nesprin-1, -2, and -3 [56]. In addition, TorA was shown to interact with SUN1 and SUN2 in a heterologous system [48], while its localization to the NE was found to be SUN1-dependent [65]. Furthermore, a knockdown of TorA disrupted the localization of KASH proteins [66]. Interestingly, recent evidence proposed a role for Torsins in the translocation of large ribonucleoprotein (RNP) particles from the nucleus into the cytoplasm in neuromuscular junctions in *Drosophila* [67] through a pathway which mechanistically resembles nuclear egress of herpesvirus [68].

For PrV, we recently demonstrated that expression of the luminal SUN2 domain, which was described to disturb normal function in a dominant-negative (dn) manner [64], resulted in lower virus titers, a severe dilation of the PNS and the ER, and an escape of primary enveloped virions from the PNS into the ER [69]. Since this was similar to the effect reported for TorA overexpression on HSV-1 [54], we were interested to study the function of TorA and B in PrV infection. Here, we overexpressed GFP-tagged wild type or mutant proteins and used the CRISPR/Cas9 genome editing system for generation of cell lines lacking TorA, TorB and TorA/B to examine how modulation of their expression affects PrV replication with special focus on nuclear egress.

## 2. Material and Methods

### 2.1. Cells and Virus

Rabbit kidney cells (RK13, CCLV-Rie 109) were cultivated in Dulbecco’s modified Eagle’s minimum essential medium supplemented with 10% fetal calf serum, provided by the Friedrich-Loeffler-Institute bio bank (Greifswald, Insel Riems, Germany). PrV strain Kaplan (PrV-Ka) [70] was propagated on RK13 cells. RK13 cells were used throughout this study since (I) they propagate PrV to high titers; (II) are easy to transfect; (III) tolerate a wide panel of foreign protein expression; and (IV) are intensively studied in our laboratory for many years.

### 2.2. DNA Constructs

SS-EGFP-TorA_WT_, SS-EGFP-TorA_ΔE302/303_, SS-EGFP-TorB_WT_, and SS-EGFP-TorB_E178Q_ constructs used in this work had been described [37,44,52,57]. Plasmid pDsRed2-ER was purchased from Takara Bio Europe, Inc. Constructs used to perform CRISPR/Cas9-mediated genome editing were generated as follows. Guide RNAs (gRNAs) were designed by targeting the first exon of TorA (*TOR1A*) or TorB (*TOR1B*) as predicted in the rabbit genome OryCun2.0 (*Oryctolagus cuniculus*, ensemble.org [71]) with the help of the online tool (http://crispr.mit.edu/). Four gRNAs with the highest score and the lowest probability for off-target effects were selected for each gene (Table 1). gRNAs were ordered as unmodified DNA oligonucleotides (MWG Eurofins, Ebersberg, Germany) with BbsI restriction overhang, hybridized and inserted into the BbsI-digested vector pX330-NeoR (kindly provided by Dr. W. Fuchs), which is a modified version of pX330-U6-Chimeric_BB-CBh-hSpCas9 (Addgene, Watertown, MA, USA, #42230) carrying an additional expression cassette for a G418 resistance for selection (previously described in Hübner, et al. [72]). The correct cloning of gRNAs was verified by Sanger sequencing at the Friedrich-Loeffler-Institut with HU6-F primer (5′-ATAATTTCTTGGGTAGTTTGCAG-3′).

### 2.3. Transfection of Cells for Co-Localization Studies

RK13 cells were seeded on coverslips in a 24-well dish and transiently co-transfected by calcium phosphate-precipitation [73] with an ER marker protein plasmid (pDsRed2-ER, Takara Bio Europe Saint-Germain-en-Laye, France) and plasmids expressing the GFP-tagged constructs. We used the calcium phosphate-coprecipitation method, although it is not very efficient, because it is milder to the cells and therefore allows to capture qualitative images later on.

### 2.4. Immunoblotting

Cells were transfected with 1 µg of plasmid DNA using polyethylenimine (PEI) [74], and harvested 24 h post transfection by scraping into the medium, pelleted, washed twice with phosphate-buffered saline (PBS) and lysed in SDS-containing sample buffer (0.13 M Tris-HCl, pH 6.8; 4% SDS; 20% glycerin; 0.01% bromophenol blue; 10% 2-mercaptoethanol). Here we used PEI transfection, instead of calcium phosphate-precipitation method, because PEI transfection is more efficient. Proteins were separated in SDS 10% polyacrylamide gels and after transfer to nitrocellulose membranes, blots were probed with a rabbit anti-GFP serum (kindly provided by Dr. G. M. Keil, FLI, Insel Riems, Germany) and a monoclonal antibody specific for alpha-tubulin (Sigma-Aldrich, Munich Germany, T5168) as loading control. After incubation with secondary peroxidase-labelled antibodies and substrate (Clarity ECL western Blot substrate, Bio-Rad, Feldkirchen, Germany), chemiluminescence was recorded in a Bio-Rad Versa Doc imager.

### 2.5. Generation of Stably Expressing RK13 Cell Lines

**Figure 1:**
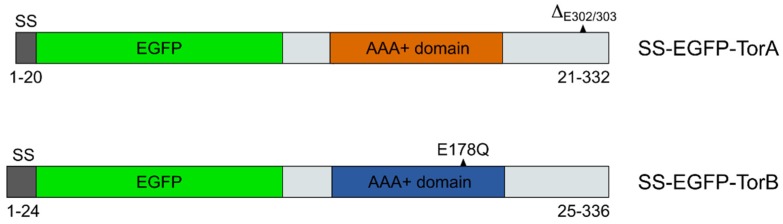
Schematic depiction of expression constructs used in this study. All constructs carried EGFP at the N-terminus. Mutations resulting in loss-of-function in TorA and B are indicated. Numbers given below represent the corresponding amino acid residues of the Torsins in the constructs used. SS: signal sequence.

For generation of cells stably overexpressing wild type or mutant forms of Torsins A and B, cells in a 6-well dish were transfected by calcium phosphate-coprecipitation [73] using 1.5 µg of plasmid DNA expressing protein constructs schematically depicted in Figure 1. Two days after transfection, cells were transferred to 10 cm plates (Corning, Kaiserslautern, Germany) and selected in medium containing 500 µg/mL G418 (Invitrogen, Schwerte, Germany). Ten to 14 days after transfection GFP-positive cell colonies were picked by aspiration and further analyzed. Cell clones were seeded on cover slips in a 24-well plate for analysis of protein localization.

### 2.6. Generation of Stable RK13 Knockout Cell Lines

Stable KO cell lines were generated by co-transfection of all four gRNA-containing pX330-NeoR constructs (1.5 µg per plasmid) using calcium phosphate-co-precipitation [73]. For DKO, all eight plasmids were co-transfected simultaneously. Two days after transfection in 6 well dishes, cells were transferred to 10 cm plates (Corning) and selected in medium containing 500 µg/mL G418 (Invitrogen). Ten to 14 days after transfection cell colonies were picked by aspiration and tested for KO by sequencing of the targeted gene sequence.

### 2.7. Test for Bi-Allelic Gene Knockout

DNA of the potential KO cell clones was isolated using Bradley Lysis Buffer (10 mM Tris, 10 mM EDTA, 0.5% SDS, 10 mM NaCl) with Pronase (1 mg/mL) and following ethanol precipitation. The targeted gene region was amplified with Phusion^®^ High-Fidelity DNA Polymerase (NEB, Frankfurt am Main, Germany) and primers given in Table 2. The gel purified phosphorylated PCR products were then blunt-end cloned into EcoRV-digested and dephosphorylated pBluescript II SK (+) (Stratagene, Darmstadt, Germany). Ten white colonies each were randomly picked [75], plasmid DNA was isolated and sequenced using the vector specific T7 primer by Sanger sequencing. In cases where all ten sequenced plasmids carried identical inserts, plasmids of five additional bacterial clones were isolated and sequenced. Mutations induced by Cas9 nuclease were identified by nucleotide sequence alignments with the rabbit genome (OryCun2.0) using Geneious 11.1.5 (https://www.geneious.com).

### 2.8. PrestoBlue Assay

Cell viability of modified and knockout cells was determined using Presto Blue™ Reagent (Thermo Scientific, Dreieich, Germany), a resazurin-based metabolic assay. RK13 wild type cells were used as control. 1 × 10^4^ cells in 90 µL volume were seeded in a black 96-well plate with a flat and clear bottom (Corning). At 24, 48 and 72 h after seeding 10 µL Presto Blue Reagent was added to the cells and resuspended. The samples were incubated for 30 min at 37 °C. For each time point, cells were measured in triplicates and eight medium containing wells were included for background estimation. Before bottom-read measuring of fluorescence in a Tecan Reader at Ex560/Em590, the plate was shaken for 5 sec. Multiple reads per well (3 × 3) were performed using the i-control™ microtiter reader software. Blank-reduced raw data (fluorescence intensities) are given in the corresponding figures. For standardization, we used the following formula: (measured value – mean)/standard deviation. Statistics was applied on the standardized values.

### 2.9. In Vitro Replication Studies

To test the efficiency of PrV propagation in the generated cell lines, cells were infected with PrV-Ka at a multiplicity of infection (MOI) of 5. Cells and supernatants were harvested at different time points after infection (0, 4, 8, 12, 24 and 30 h p.i.). To determine the infectious virus titer, samples were thawed, cell debris was removed by centrifugation (2 min, 15,000 rpm), the supernatant was serially diluted (10^−1^ to 10^−6^) and used to infect RK13 cells in 24 well culture plates. After incubation for 1 h, the inoculum was replaced by a semi sold medium allowing only direct cell-to cell spread of the virus. Cells were fixed after 2 days with formaldehyde and stained with crystal violet. Virus plaques, which were detectable as holes in the blue-stained cell monolayer, were counted in at least two different wells and mean values were calculated as plaque forming units per milliliter (pfu/mL).

Shown are mean values of three (EGFP-TorA, -B overexpressing cells) or six (knockout cells) independent experiments with corresponding standard deviations. To exclude clonal and putative second site effects at least three different cell clones were tested initially for each mutated cell line.

### 2.10. Statistics

For each assay at least three independent experiments were performed. The statistical significance of the data presented in Figures 5, 6, 7 and 8 was determined by a two-way ANOVA followed by Dunnett’s multiple comparison test. All statistical tests were performed using GraphPad Prism version 8.1.0 (GraphPad Software, La Jolla, CA, USA). We compared the mean of each time point with the mean of the corresponding parental RK13 cells. A *p*-value ≤ 0.05 was considered significant and is presented in Figures 6 and 8 by the presence of asterisks (*, *p* ≤ 0.05, **, *p* ≤ 0.01, ****, *p* ≤ 0.0001).

### 2.11. Laser Scanning Confocal Microscopy

For confocal microscopy, we used stably expressing RK13 cells and RK13 cells transiently co-expressing the GFP-tagged plasmids and an ER-marker plasmid [73]. In addition, RK13 and Torsin knockout cells were infected with 250 pfu of PrV-Ka. Cells in 24 well dishes were fixed with 4% paraformaldehyde for 15 min one day after seeding for the stable expressing cells or two days after transient transfection. Infected cells were analyzed 18 h p.i. Fixed cells were washed three times and then incubated for 30 min with 50 mM NH_4_Cl in 1X PBS to quench the free aldehyde groups after PFA fixation. The GFP-tagged proteins and the DsRed-ER marker proteins were directly visualized via their autofluorescence. After permeabilization with 0.1% Triton X-100 in 1x PBS and subsequent blocking for 20 min with 0.25% skimmed milk the viral antigen was stained with a polyclonal rabbit serum specific for pUL34 (1:500, [76]). Alexa-Fluor 568-conjugated goat anti-rabbit IgG (dilution 1:1000, Invitrogen) was used to detect bound antibody. The nuclei were counterstained with 300 mM DAPI for 5 min and cells were mounted in a drop of Kaiser’s glycerol gelatin (Merck, Darmstadt, Germany). Samples were analyzed using with a confocal laser scanning microscope (Leica DMI 6000 TCS SP5, 63× oil-immersion objective, NA = 1.4; Leica, Wetzlar, Germany). Representative images were processed using the Fiji software [77,78]. Scale bars indicate 10 µm.

### 2.12. Ultrastructural Analyses

RK13 and KO cell lines were infected with PrV-Ka at an MOI of 1 for 14 h and processed for transmission electron microscopy as described previously [76]. Numbers of primary virions present in the PNS in infected RK13 and RK13-TorA/B_DKO_ were counted in 10 different sections each.

## 3. Results

### 3.1. Influence of Torsin Overexpression on PrV Replication

To test whether overexpression of either the GFP-tagged wild type or mutated forms of (human) Torsins A and B has an effect on PrV replication, the different expression constructs (Figure 1) were transfected into RK13 cells for transient expression and generation of stably expressing cell lines. Torsins are well conserved in metazoans [34] and functional expression of the same constructs in murine cells was reported [66].

**Figure 2:**
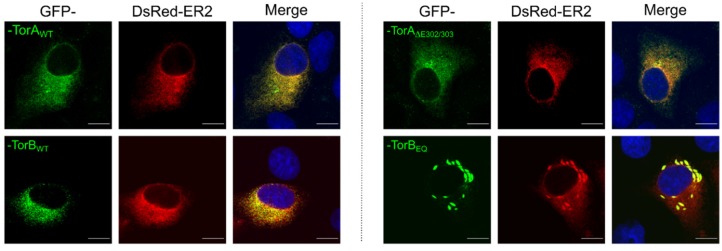
Localization of GFP-tagged constructs in RK13 cells. Representative images of RK13 cells transiently expressing the GFP-tagged constructs. Cells were co-transfected with plasmids expressing the DsRed2-ER marker and the GFP-tagged Torsins. Nuclei were counterstained with DAPI and autofluorescence was detected with a confocal laser scanning microscope. Scale bars indicate 10 µm.

**Figure 3:**
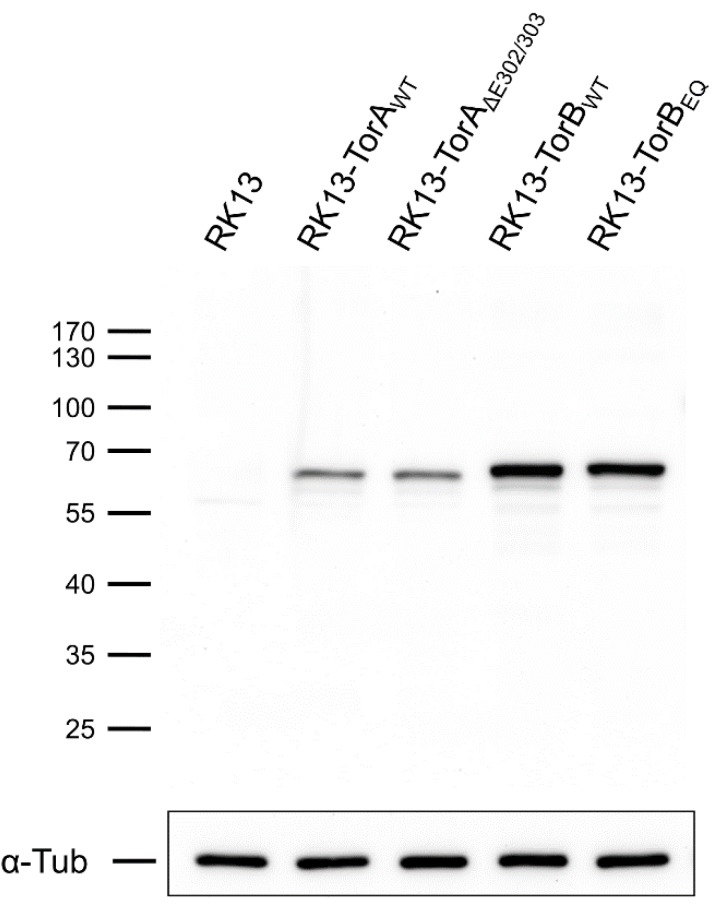
Expression of GFP-tagged Torsins in RK13 cells. Lysates of transfected cells were harvested, and proteins were separated in SDS 10%-polyacrylamide gels. Blots were probed with a GFP-specific rabbit antiserum and a monoclonal antibody against α-tubulin as loading control. Molecular masses of marker proteins (in kDa) are indicated on the left.

As expected, each of these constructs was targeted to the ER/NE when transiently expressed in RK13 cells (Figure 2) showing a clear colocalization with the DsRed2-tagged ER marker (DsRed2-ER) [38,46,47]. Consistent with previous reports, the expression of SS-EGFP-TorB_E178Q_ resulted in the appearance of dense protein accumulations within the ER [46]. Furthermore, each of the proteins was expressed in RK13 cells at the predicted molecular mass evaluated in western blot analysis (Figure 3). The SS-EGFP-tagged TorA and TorB constructs were ~65–70 kDa, with expression levels of TorB slightly higher than for TorA. Taken together, these results demonstrate that the GFP-Torsin constructs are expressed properly in RK13 cells.

**Figure 4:**
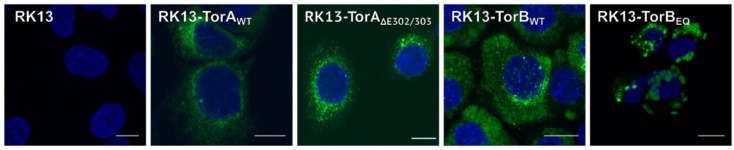
Localization of stably expressed RK13-GFP-TorA and -TorB. Representative merged images show the intracellular localization of RK13 cells stably expressing the GFP-tagged mutant and wild type proteins. Nuclei were stained with DAPI, and GFP autofluorescence was detected with a confocal laser scanning microscope. Scale bars indicate 10 µm.

**Figure 5:**
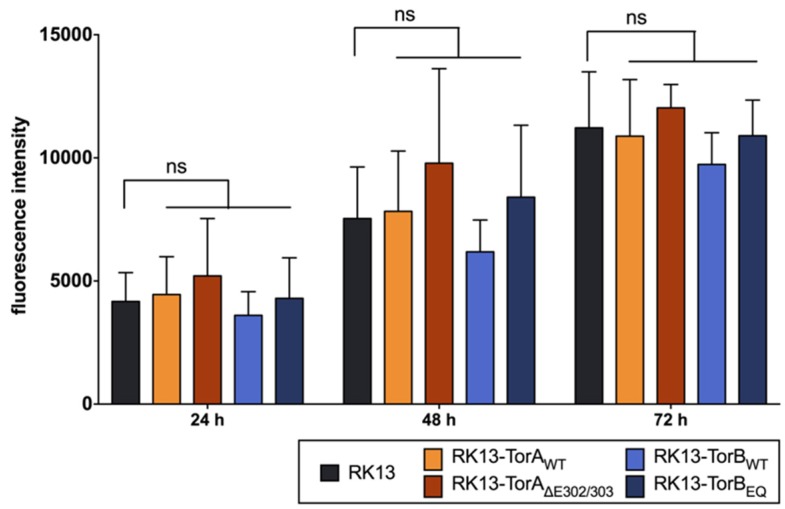
Cell viability of the modified cell lines. RK13 control and stably expressing RK13-GFP-TorA and -TorB cells were seeded with 1 × 10^4^ cells per well and at 24, 48, and 72 h post seeding the mitochondrial activity was measured (in fluorescence intensities) via the Presto Blue Assay. Shown is the mean of three independent experiments, ns = statistically not significant.

**Figure 6:**
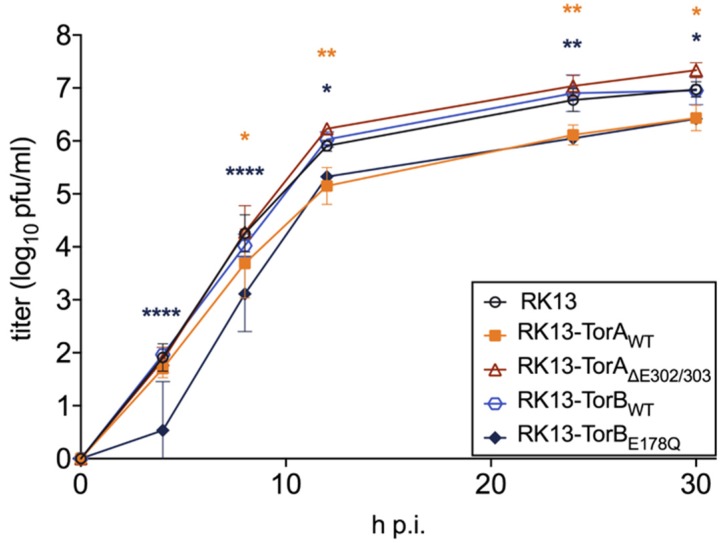
Effects of overexpression of Torsins on PrV replication. Stably expressing cell lines and parental RK13 cells were infected with PrV-Ka (MOI of 5) and harvested at different time points. Progeny virus titers were determined on RK13 cells. Given are mean values of three independent experiments with corresponding standard deviations. Statistically significant differences compared to the parental RK13 were determined by GraphPad Prism software and are indicated by asterisks in the same color as the corresponding graphs (*, *p* ≤ 0.05, **, *p* ≤ 0.01, ****, *p* ≤ 0.0001).

Stably expressing cell lines were selected for homogeneous GFP expression. The subcellular localization of each protein in these cell lines was indistinguishable from what was observed in transient expression (Figure 4). Ultrastructurally, cells overexpressing TorB showed a significant expansion of the rough ER with either diffuse matter (TorB_WT_) or filled with protein filaments (TorB_E178Q_) (data not shown) but no sinusoidal ER structures as reported for HeLa cells [46]. No deleterious effects on cellular metabolic activity were observed from overexpression of the GFP-tagged cellular genes (Figure 5). To test whether overexpression of these constructs influences virus replication, stably expressing cells as well as parental RK13 cells were infected with PrV strain Kaplan (PrV-Ka) [70] at an MOI of 5 and harvested at different time points after infection. As shown in Figure 6 small but significant 3- to 5-fold titer reduction was found after infection of RK13-TorA_WT_ at all time points later than 8 h after infection, while cells expressing the mutant form TorA_∆E302/303_ supported PrV replication to similar titers as non-transgenic RK13 cells. For RK13-TorB_WT_ cells there were no significant changes in viral titers compared to parental RK13 cells, while infection of TorB_E178Q_ expressing cells resulted in 4 to 7-fold titer decrease at all time points after infection.

### 3.2. Torsin A and Torsin B Are Required for Efficient PrV Replication in RK13 Cells

**Figure 7:**
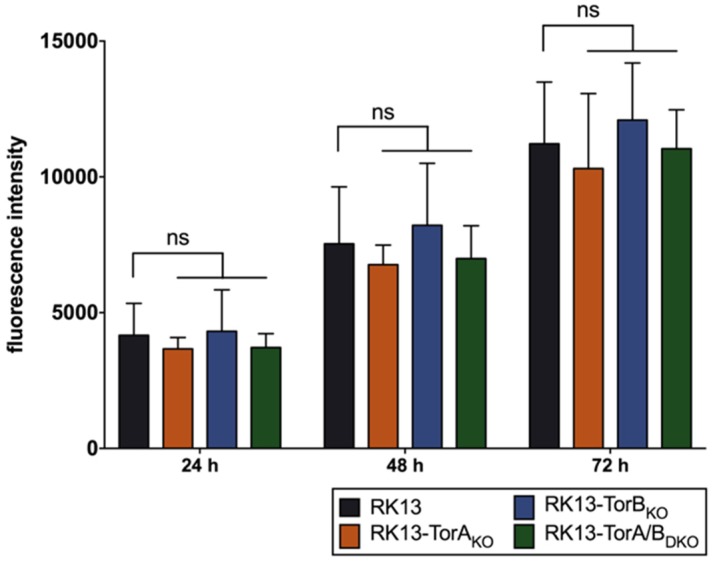
Cell viability of generated modified cell lines. RK13 control together with Torsin_KO_ cell lines were seeded with 1 × 10^4^ cells per well and at 24, 48, and 72 h post seeding the mitochondrial activity was measured (in fluorescence intensities) with the Presto Blue Assay. Shown is the mean of three independent experiments, ns = statistically not significant.

Torsin A and B are suggested to be functionally redundant [52]. Surprisingly, overexpression of the wild type TorA and the mutant TorB_E178Q_ slightly impaired PrV replication while the other two forms had no significant impact pointing to different mechanisms. We were interested to analyze whether this effect might be more pronounced when both proteins are targeted simultaneously. Since equivalent simultaneous expression of both proteins in cell lines is difficult to achieve and maintain, we decided to use the CRISPR/Cas9 genome editing system to generate single and double KO cell lines for TorA and TorB. Four guide RNAs per gene were designed (Table 1), cloned into vector pX330-NeoR [72], and transfected simultaneously into RK13 cells. Genomic DNA of several cell clones was isolated, and the target region was amplified by PCR using primers given in Table 2. The PCR products were cloned into pBluescript SK+ and plasmid DNA from at least ten bacterial colonies each was isolated and sequenced. Wild type sequences in comparison to the mutations found in the different plasmids are summarized in Table 3. All cloned PCR products derived from RK13-TorB_KO_ and RK13-TorA/B_DKO_ exhibited only a single type of mutation indicating that both alleles carry the same deletion. Two different allelic variants were present in RK13-TorA_KO_ cells. In-frame deletions were found in the TorB allele of RK13-TorA/B_DKO_ cells (∆30 bp, both alleles). Unfortunately, the tested antisera, which are specific for human Torsins, did not detect the corresponding homologs in RK13 cells (data not shown). As similar to the stably expressing cells described above, no deleterious effects on metabolic activity were observed in the KO cell lines (Figure 7).

**Figure 8:**
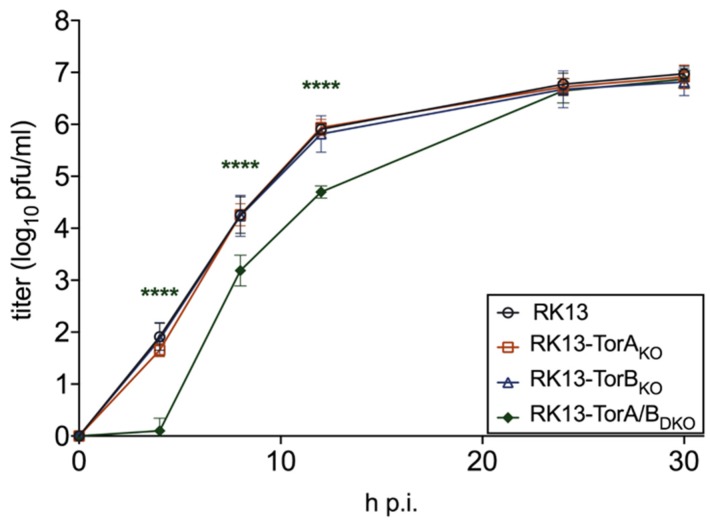
Effect of gene knockout on PrV replication. RK13 and Torsin_KO_ cell lines were infected with PrV-Ka (MOI of 5) and harvested at different time points after infection. Progeny virus titers were determined on RK13 cells. Shown are mean values of six independent experiments. Statistics were done with GraphPad Prism software and asterisks indicate statistically significant differences compared to the parental RK13 in the same color as the corresponding graph (****, *p* ≤ 0.0001).

The generated KO and DKO cell lines were tested for virus propagation by infection with PrV-Ka at an MOI of 5 and harvested at different times after infection. As shown in Figure 8, no significant differences in viral progeny titers were found for RK13-TorA_KO_ and RK13-TorB_KO_ cells. In TorA/B_DKO_ cells a significant drop in virus titer compared to the parental RK13 cells was observed at 4, 8 and 12 h after infection which disappeared at later time points.

**Figure 9:**
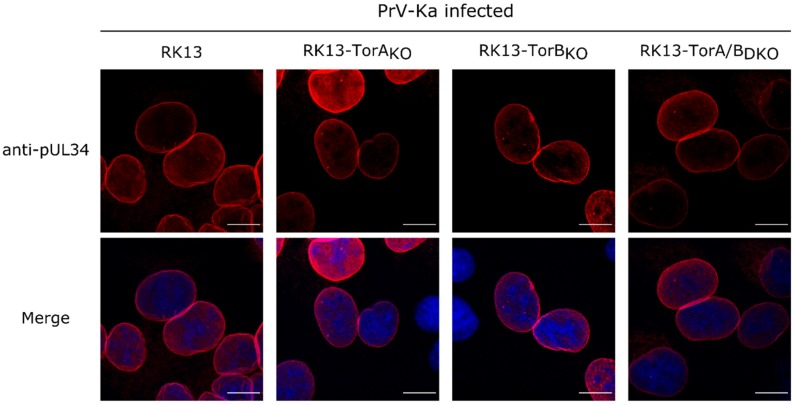
Localization of the NEC component pUL34 in PrV-Ka infected RK13 and Torsin_KO_ cells. Representative images showing undisturbed localization of the NEC component pUL34 (red) in infected RK13-Torsin_KO_ cells. Nuclei were counterstained with DAPI (blue) and the fluorescence was imaged with a confocal laser scanning microscope. Scale bars indicate 10 µm.

To test whether the absence of Torsin A and/or B influences nuclear envelope localization of the viral NEC component pUL34, parental RK13 and Torsin knockout cells were infected with PrV-Ka under plaque assay conditions. Cells were fixed after 18 h and stained with the monospecific anti-pUL34 rabbit serum [76]. As shown in Figure 9, no difference in nuclear rim staining for pUL34 was obvious independent of presence or absence of Torsins A, B or both proteins.

**Figure 10:**
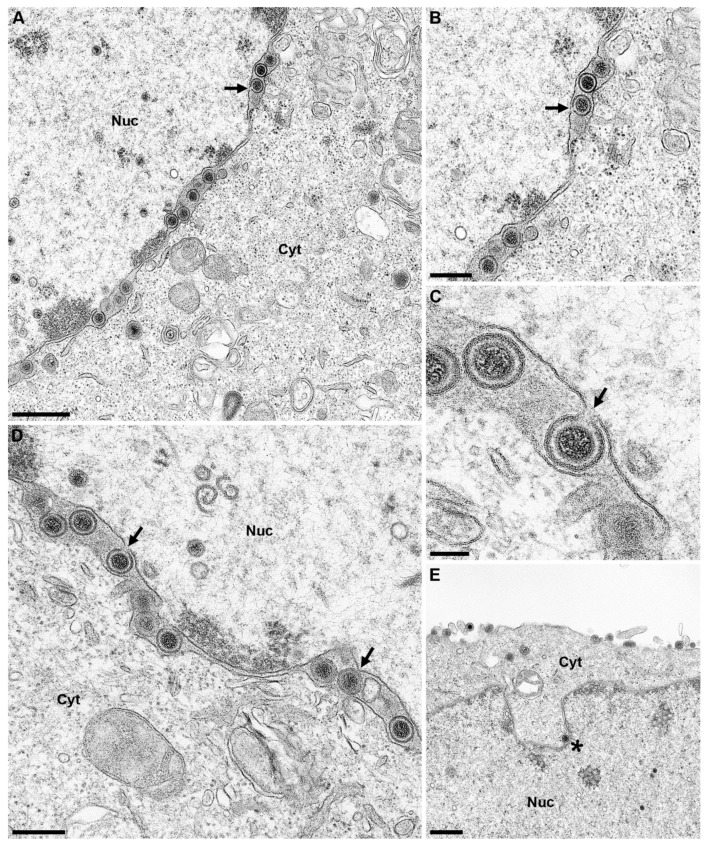
TorA/B DKO results in accumulation of primary enveloped virions in the PNS. RK13, RK13-TorA_KO_, RK13-TorB_KO_ and RK13-TorA/B_DKO_ cells were infected with PrV-Ka (MOI of 1) and processed for electron microscopic imaging 14 h p.i. No obvious effect was found for the single KO cells (data not shown), while the TorA/B_DKO_ showed accumulations of primary virions within the PNS, many of them still connected to the INM by a short neck (indicated by arrows) (**A**–**D**). Panel (**B**) shows a higher magnification of the infected cell in panel (**A**), while panel (**C**) shows a higher magnification of panel (**D**). Panel (**E**) shows a rare case of a primary virion in the PNS in parental RK13 cells (marked by asterisk). Scale bars indicate 600 nm in panel (**A**,**E**), 300 nm in panel (**B**,**C**) and 100 nm in panel (**D**). Nuc: nucleus, Cyt: cytoplasm.

Although no drastic effect on viral titers was observed, we analyzed nuclear egress at the ultrastructural level. All KO cell lines were infected with PrV-Ka at an MOI of 1 and processed for electron microscopy. We did not observe any impairment in nuclear egress or virion morphogenesis in infected RK13-TorA_KO_ and RK13-TorB_KO_ (data not shown). In contrast to previous studies [45,51,52,53], nuclear envelope blebbing in the Torsin KO cells was not obvious. However, in PrV infected RK13-TorA/B_DKO_ cells primary enveloped virions accumulated in the PNS which is in contrast to PrV infected parental RK13 cells, exhibiting only rare single virions in the PNS (Figure 10E; marked by an asterisk). We counted the number of primary enveloped virions in 10 sections each of PrV-Ka infected RK13 and RK13-TorA/B_DKO_ cells. While in RK13 cells 5 primary enveloped virions could be detected in 30 nuclei, infected RK13-TorA/B_DKO_ cells contained 593 primary virions in 52 nuclei. Compared to the accumulations observed in mutants lacking the pUS3 protein kinase [15], primary virions did not preferentially accumulate in herniations of the INM but were mainly found lined up in the PNS (Figure 10). Fission from the INM seemed to be less efficient in the absence of TorA/B since primary virions were frequently found still attached to the INM by a small neck (Figure 10, arrows).

## 4. Discussion

Herpesvirus nucleocapsids rely on a vesicular pathway engaging the NE for nuclear egress. While the viral NEC orchestrates budding at and scission from the INM, no viral protein essential for de-envelopment at the ONM could be identified yet, while the pUS3 protein kinase exhibits a regulatory role (reviewed in Mettenleiter, Klupp and Granzow [5], Mettenleiter, Muller, Granzow and Klupp [7], and Johnson and Baines [6]).

Here, we analyzed a possible converging role for Torsin in herpesvirus nuclear egress by overexpressing them singly, and by generation of single and double KO cells using CRISPR/Cas9 based mutagenesis in the rabbit kidney cell line RK13. Torsins A and B seem to be ubiquitously expressed and are at least partially functionally redundant, complicating interpretation of experimental data generated by targeting only one form. Overexpression of TorA_WT_ or TorB_E178Q_, which carries a mutation rendering the protein unable to hydrolyze ATP [46], resulted in a small but significant drop in virus titers compared to the non-transgenic RK13 control. A similar drop in virus titer was also observed for HSV-1 after infection of a neuronal cell line expressing TorA_WT_ [54]. Primary enveloped virions escaped into the lumen of the ER as it was shown after overexpression of a dominant-negative SUN2 [69]. These data support the notion that Torsins regulate the SUN/nesprin interaction and that an intact LINC complex/Torsin relationship is necessary to restrict primary virions in the PNS [54]. However, neither TorA_WT_ nor TorB_E178Q_ expression resulted in an obvious increase of the number of primary virions in cytoplasmic structures, alteration of the nuclear envelope or the spacing between the INM and ONM (data not shown). Due to the lack of antibodies detecting SUN proteins in RK13 cells, an influence of Tor expression on the LINC could not be tested.

We also generated single and double knockout cells by CRISPR/Cas9 mutagenesis, which was verified by sequencing of the corresponding gene regions. No obvious differences in growth or metabolic activity were observed between the modified cell lines stably expressing the respective Torsin constructs or in Torsin KO cell lines, compared to parental RK13 cells. In addition, we did not detect any morphological defects, indicating that the targeted proteins are non-essential for cellular proliferation under our cell culture conditions. Since we could not exclude second site effects, we always included different cell clones for each KO in the preliminary screens. In none of the KO cell lines wild-type sequences could be identified. Unfortunately, the available antisera against the corresponding proteins of human origin did not react with the rabbit homologs in the RK13 cell lysates. The used gRNAs were designed to target exons that are shared by all transcript variants of the gene of interest to minimize the chance that shorter but still functional protein isoforms might be expressed. Although the RK13-TorA/B_DKO_ cell line carries an in-frame deletion (aa 76–85) upstream of the Walker A motif (aa 105–112) in both alleles of the TorB gene and we cannot exclude expression of a truncated protein, the observed effects indicate a significant loss of function.

In contrast to the overexpression experiments, in which a slight but significant drop in progeny virus titers was found after infection of RK13-TorA_WT_ and RK13-TorB_EQ_ cells_,_ none of the single KO cells showed a significant effect on infectious virus production or on localization of the NEC component pUL34. In ultrastructural analyses, we could not detect perturbations of the NE in cells lacking TorA or B, and primary virions were only rarely detected in the PNS or in cytoplasmic vesicles arguing against an impairment of nuclear egress. However, after infection of RK13-TorA/B_DKO_ cells a drop in virus titer occurred at early time points after infection. High-resolution imaging revealed striking accumulations of primary enveloped virions lined-up in the PNS, while accumulations in herniations of the ONM were only rarely detected. Many of the primary envelopes seemed to be connected to the INM indicating that in the absence of Torsin A and B scission might be impaired (Figure 10). In line with this, in a HeLa cell line where all four known Torsins had been eliminated simultaneously, NE blebs still connected to the INM had been described [50]. Unfortunately, these cell lines were not tested for effects on herpesvirus nuclear egress. It is tempting to speculate that not only Torsin A and B, but also other Torsins might be involved in this process. Incomplete scission of primary HSV-1 virions from the INM was also reported after depletion of ESCRT-III proteins [24]. It might be speculated that recruitment of an AAA+ ATPase (Torsin/Vps4) might alleviate the scission process mediated by the NEC proteins.

It should be noted that we did not observe clear blebs of the INM into the PNS of our TorA/B_DKO_ cells, which contrasts previous reports from fibroblasts [52], HeLa cells [50], or neurons [53].

Torsins are supposed to function as regulators of the LINC complex [56] and an intact LINC complex may be required for efficient nuclear egress by restricting primary virions to the PNS in close proximity to the NE for efficient fusion of the primary envelope with the ONM [69]. In our TorA/B_DKO_ cells no impact on spacing between INM and ONM was apparent indicating no impairment of LINC.

In summary, we demonstrate that Torsins A and B, which might be involved in proper functioning of the LINC complex, play a role during nuclear egress of herpesvirus capsids. These results together with our previous findings on SUN2 impairment highlight the importance of this complex for vesicle-mediated herpesvirus capsid transport through the nuclear envelope.

## Figures and Tables

**Table 1:** Oligonucleotide sequences. Compatible 5′ overhangs for restriction enzyme BbsI used for cloning are underlined.

Name	Sequence (5′–3′)
TorA_gRNA#1_Fwd	CACCCTGGCGGTAGCGCCGGTCGG
TorA_gRNA#1_Rev	AAACCCGACCGGCGCTACCGCCAG
TorA_gRNA#2_Fwd	CACCTGTCTGGCGGTAGCGCCGGT
TorA_gRNA#2_Rev	AAACACCGGCGCTACCGCCAGACA
TorA_gRNA#3_Fwd	CACCCGCCGGTCGGTGGTCAGCGC
TorA_gRNA#3_Rev	AAACCGCTGACCACCGACCGGCGG
TorA_gRNA#4_Fwd	CACCGTTCCTGCGCTGACCACCGA
TorA_gRNA#4_Rev	AAACGTCGGTGGTCAGCGCAGGAA
TorB_gRNA #1_Fwd	CACCGTGATTCTGAAGGCGCTGAC
TorB_gRNA #1_Rev	AAACGTCAGCGCCTTCAGAATCAC
TorB_gRNA #2_Fwd	CACCCGCCTTCAGAATCACTTCCG
TorB_gRNA #2_Rev	AAACCGGAAGTGATTCTGAAGGCG
TorB_gRNA #3_Fwd	CACCTTTTTGGTTTTTGGTAACGA
TorB_gRNA #3_Rev	AAACTCGTTACCAAAAACCAAAAA
TorB_gRNA #4_Fwd	CACCGAAGCTGTTCGGACAGCATC
TorB_gRNA #4_Rev	AAACGATGCTGTCCGAACAGCTTC

**Table 2:** Primers used for amplification of targeted gene.

Name	Sequence (5′–3′)
TorA_seq_Fwd	CACCGGAGACAGCTATAGCC
TorA_seq_Rev	GACCTTCTTGGCCAGATGCT
TorB_seq_Fwd	CCGCGCGAATGTGAAGTGCGCCCCCGTGGAAC
TorB_seq_Rev	GTCTTGTGCTCATGCGGGAAGTGCAGTGTG

**Table 3:** Summary of mutations detected in the KO cell lines.

Mutant		Genotype	Sequence	Mutations
TorA_KO_		wild type	GAACCGGAAGAGCGTGTCTGGCGGTAGCGCCG..GTCGGTGGTCAGCGCAGGAAGGCGCGGGGAGGCG	
		knockout	GAACCGGAAGAGCGTGTCTGGCGGTAGCGC**G**GTGGT----GGTCAGCGCAGGAAGGCGCGGGGAGGCG (3) GAACCGGAAGAGCGTGTCTGGCGGTAGCGCCG..GT----GGTCAGCGCAGGAAGGCGCGGGGAGGCG (7)	1 nt Ex, 2 bp In, 4 bp Del4 bp Del
TorB_KO_		wild type	CTTGGAGAAGCTGTTCGGACAGCATCTGGCCACGGAAGTGATTCTGAAGGCGCTGACCGGCTTCAAGA	
		knockout	CTTGGAGAAGCTGTTCGGACAGC-----------------------------CTGACCGGCTTCAAGA (15)	29 bp Del
TorA/B_DKO_	TorA	wild type	GAACCGGAAGAGCGTGTCTGGCGGTAGCGCCGGTCGGTGGTCAGCGCAGGAAGGCGCGGGGAGGCGCG	
		knockout	GAACCGGAAGAGCGTGTCTGGCGGTAGCGCC**C**G-------TCAGCGCAGGAAGGCGCGGGGAGGCGCG (15)	1 nt Ex, 7 bp Del
	TorB	wild type	CTTGGAGAAGCTGTTCGGACAGCATCTGGCCACGGAAGTGATTCTGAAGGCGCTGACCGGCTTCAAGA	
		knockout	CTTGGAGAAGCTGTTCGGACA------------------------------GCTGACCGGCTTCAAGA (15)	30 bp Del

The sequence for each targeted gene region was compared to the sequence of the parental RK13 sequence. Numbers in brackets indicate the frequency of InDel (Insertion or Deletion) mutations found in the clones sequenced. Deletion (Del) of a base pairs (bp) is shown as hyphen, insertion (In) of a bp is marked by a dot in the parental sequence and nucleotide (nt) exchanges (Ex) are shown in bold characters.
